# Managing Successional Stage Heterogeneity to Maximize Landscape-Wide Biodiversity of Aquatic Vegetation in Ditch Networks

**DOI:** 10.3389/fpls.2018.01013

**Published:** 2018-07-16

**Authors:** Sven Teurlincx, Michiel J. J. M. Verhofstad, Elisabeth S. Bakker, Steven A. J. Declerck

**Affiliations:** ^1^Department of Aquatic Ecology, Netherlands Institute of Ecology (NIOO-KNAW), Wageningen, Netherlands; ^2^The Dutch Botanical Research Foundation (FLORON), Nijmegen, Netherlands

**Keywords:** aquatic plants, diversity partitioning, beta-diversity, cyclic rejuvenation, species replacement, richness difference, drainage ditch, macrophytes

## Abstract

The presence of a high diversity of different successional stages in a landscape may help to conserve and promote landscape-wide biodiversity. A strategy to achieve this is using Cyclic Rejuvenation through Management (CRM), an approach employed in a variety of different ecosystems. CRM periodically resets the successional stages in a landscape. For aquatic systems this constitutes vegetation removal and dredging. For this approach to be useful (a) successional stages are required to be different in community composition and (b) these differences need to be caused by true replacement of species between stages. While potentially valid, these assumptions are not generally tested prior to application of CMR. In this study we test these assumptions to explore the usefulness of managing on successional stage heterogeneity for maximizing landscape-wide aquatic plant diversity. We carried out vegetation surveys in the ditch networks of 21 polder landscapes in Netherlands, each containing 24 ditch reaches. Using a clustering approach combined with insight from literature on vegetation succession in these systems we assigned our sampled communities to defined successional stages. After partitioning landscape diversity into its alpha and beta components, we quantified the relative importance of replacement among successional stages. Next, through scenario analyses based on simulations we studied the effects of reducing successional stage heterogeneity on landscape-wide biodiversity. Results showed that differences in community composition among successional stages were a potentially important factor contributing to landscape diversity. Early successional stages were characterized by higher replacement of species compared to late successional stages. In a scenario of gradual decrease of heterogeneity through the systematic loss of the earliest successional stages we found 20% of the species richness in a polder was lost, pointing toward the importance of maintaining early successional stages in a polder. This makes a compelling case for application of CRM within agricultural drainage ditch landscapes to maximize regional aquatic plant diversity. While applied to drainage ditch systems, our data-driven approach is broadly applicable to other systems and may help in providing first indications of the potential of the CRM approach. We argue that CRM may maintain and promote regional biodiversity without compromising the hydrological function of the systems.

## Introduction

Land use intensification and global change have led to decreasing biotic diversity (Foley et al., 2005). Global species numbers show clear negative trends, though different levels of spatial scale show very different trends (McGill et al., 2015). Much of this biodiversity loss is caused by increasing homogenization of communities (i.e., biotic homogenization), and not necessarily by loss of local diversity (Dornelas et al., 2014). For the landscape scale this may imply that landscape diversity may decrease mainly due to the disappearance of differences between local communities (Smart et al., 2006), rather than locally detectable declining species numbers (McGill et al., 2015). As declines in biodiversity threaten the multifaceted functioning of ecosystems at both local and landscape scales (van der Plas et al., 2016), its conservation and restoration requires appropriate landscape-wide management strategies.

The process of ecological succession has long been acknowledged as a primary driver of biodiversity (Sousa, 1979). Different stages of ecological succession may exhibit different species richness levels. A classic example on forest succession shows higher species numbers in intermediate stages of succession (Odum, 1969; Whittaker, 1970). Furthermore, different successional stages may harbor very different sets of species. Thus, the landscape diversity is not a function of the local diversity alone, but also of the complementarity between stages present in the landscape. Hence, to maximize the diversity of a landscape, both the local diversity of successional stages, as well as the difference between communities of the different stages needs to be considered. This line of thought is well represented in the classical partitioning of the landscape diversity (γ) into a local component (α) and a turnover component (β) (Whittaker, 1960; Jost et al., 2010). Periodic resets of unidirectional succession through naturally occurring disturbance events in different parts of the landscape may contribute to the maintenance of a mosaic of successional stages (Sousa, 1984). In natural systems, examples of such disturbances include fire (Vandvik et al., 2005), scouring by peak river discharges (Tockner et al., 2000) or landslides (Walker et al., 1996). In absence of such natural dynamics, for example due to human interventions, there is a risk of loss of successional stage heterogeneity within the landscape (Baptist et al., 2004).

To obtain biodiversity within a landscape that has lost its natural dynamics due to human influence, management efforts need to be directed toward maintaining a landscape with a variety of successional stages present. Such management has been widely applied in a variety of different ecosystems (Baptist et al., 2004; Vandvik et al., 2005) and is known by different names, e.g., cyclic rejuvenation, rotational management and periodic ecosystem reset. Here we use the term cyclic rejuvenation through management (CRM), which is the practice of periodically resetting part of the habitat in a landscape to a (mostly) bare state in order to create a spatially dynamic mosaic of habitat patches in different stages of succession (Hinsch and Poethke, 2007). CRM in floodplain management, aimed at systematic removal of part of the floodplain forests, increased biodiversity in channels where natural reset of succession was absent (Baptist et al., 2004). Likewise, controlled fire management can help preserve grassland diversity through CRM (Richards et al., 1999). While good results have been shown, the inherent success of this management approach rests strongly on the assumption that successional stages are complementary to one another. The more unique stages are with respect to their community composition, the larger the gain to the regional species pool and thus overall landscape diversity. Conversely, when successional stages are highly similar, CRM will have little effect on landscape diversity.

Manmade water systems such as agricultural drainage ditches are a good example of anthropogenic ecosystems which are under continuous management to protect the hydrological drainage of agricultural land (Hill et al., 2016). However, their value for conservation of biodiversity is increasingly recognized as well (Armitage et al., 2003; Herzon and Helenius, 2008; Clarke, 2015). Reshaping ditch banks, removing vegetation and dredging organic sediment helps to maintain hydrological functioning, but may also support ecological function and diversity (Twisk et al., 2003). Conventional management of these waterways results in the constant resetting of succession (van Strien et al., 1991; Clarke, 2015; Hill et al., 2016), making an often unorganized form of CRM the norm in these systems. Formal CRM potentially offers an approach for this management to take place, while also realizing a biodiversity increase in comparison to existing management.

In human dominated landscapes, management primarily aims at maintaining provisioning services [e.g., water storage, food production (Power, 2010)]. For ditch ecosystems, the application of CRM has been suggested as a promising way to increase diversity at a landscape scale (Watson and Ormerod, 2004; Clarke, 2015; Hill et al., 2016) while maintaining associated services. Landscape level diversity (γ) in these ecosystems has been shown to be largely caused by differences in community composition between individual sites (Goldenberg Vilar et al., 2014; Whatley et al., 2014a), as indicated by a large β-diversity component, stressing the importance of landscape heterogeneity. Despite the claims on the potential usefulness of CRM (Clarke, 2015), so far no formal evaluation of its potential has been made, nor has it been widely adopted in practice in ditch management. A first step in the evaluation and adoption of such a management practice is to illustrate the importance of successional stage heterogeneity for landscape-wide diversity. Differences in community composition between sites may be caused by two inherently different underlying patterns, namely one of species richness difference and one of species replacement (Baselga, 2010; Legendre, 2014). Differences in community composition between sites (β-diversity) may be partitioned into a richness difference and species replacement component (Baselga, 2010; Podani and Schmera, 2011; Legendre, 2014). Species replacement refers to the simultaneous gain and loss of species along an ecological gradient (Legendre, 2014). A large replacement component is indicative for high levels of complementary between sites in terms of species composition. Alternatively, compositional differences among sites may also be generated merely by differences in species numbers (richness difference, *sensu* Legendre, 2014, also see Podani and Schmera, 2011). In contrast to the latter, it is the replacement component that will contribute to γ-diversity. Translating this to ditch networks, the degree to which successional stage diversity will be important in generating a high γ-diversity at the landscape scale will largely depend on the relative importance of the replacement component in the β-diversity among successional stages. For CRM to truly be useful for increasing landscape biodiversity, species replacement between different successional stages is required (i.e., complementarity) and not difference in richness alone.

In this study we performed 504 vegetation surveys in ditch reaches spread over 21 different polder landscapes in the Netherlands to study the importance of maintaining successional stage diversity on the landscape-wide species diversity of aquatic vegetation. We characterized the dissimilarity among successional stages and its underlying patterns to assess the complementarity of communities of different successional stages and their contribution to landscape biodiversity. In addition, we tested the potential effect of landscape-wide heterogeneity in successional stages on the landscape-wide diversity. This was done by calculating the biodiversity of simulated landscapes with different combinations of successional stages. Based on these results we evaluate the hypothesized merit of CRM on promoting landscape-wide diversity in ditch systems (Clarke, 2015) through proliferation of a diverse spatial-temporal mosaic of habitats.

## Materials and Methods

### Study Landscapes and Site Selection

**FIGURE 1:**
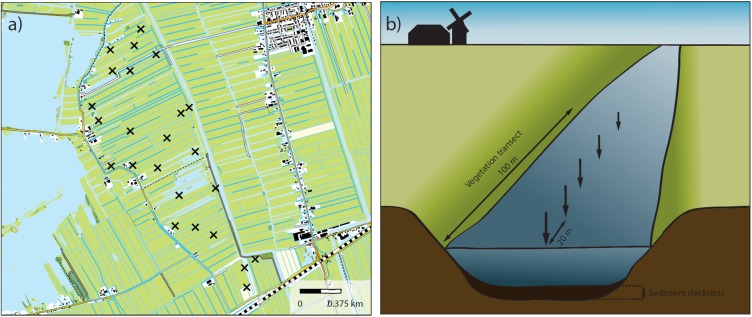
**(a)** Example of one of the 21 studied polder landscapes. Crosses indicate the predetermined locations where ditch reaches were sampled. **(b)** Illustration of the ditch reach with the vegetation transect (100 m) and the five points at which organic sediment thickness was measured.

Our study took place in the peat meadow polder landscapes in the west of Netherlands (coordinates given in Supplementary Table S1). These landscapes were historically created by drainage of peatlands to reclaim land for agriculture. This has led to the typical Dutch polder landscapes in which long, narrow fields are intersected by a network of drainage ditches, with the whole system being surrounded by dykes. Water level fluctuations within these landscapes are strictly controlled. The ditches in these systems are generally eutrophic to hypertrophic due to their long time agricultural use. The fields are used primarily for intensive cattle farming, although some variation in land use intensity is generated by governmental subsidies (agri-environmental schemes) that stimulate farmers to cultivate land less intensively (Catry et al., 2017). Additionally, some fields are owned by nature management organizations and managed more extensively with the aim of creating and sustaining moist natural grassland vegetation and its associated biota. In all cases the ditches need to be regularly managed to sustain their hydrological function. This is done through removal of plant biomass (e.g., mowing) and dredging of organic sediments from the ditch bottom. In our study we selected 21 different polder landscapes of roughly 200 hectares each. Agricultural land use intensity in these landscapes varied greatly, ranging from relatively extensive (low density, organic dairy farming, nature conservation area) to very intensive (e.g., high density cattle stocks). Within each of these 21 landscapes we selected 24 ditch reaches. Each landscape was first divided into 24 equal subparts. Within each of these subparts one ditch reach of 100 m length was selected randomly. In this way, reaches were spread more or less evenly across each landscape while their exact location was still selected on a random basis within the confines of each subpart (**Figure 1a**). Vegetation surveys were carried out along each ditch reach and the relative abundance of all plant species growing in the water was assessed according to the Tansley scale (Tansley, 1946). These Tansley dominance classes were converted to cover percentages using Supplementary Table S2. Furthermore, the thickness of the organic sediment layer was measured at five locations (every 20 m) in the ditch reach in the center of the ditch (**Figure 1b**).

### Defining Successional Stages

**FIGURE 2:**
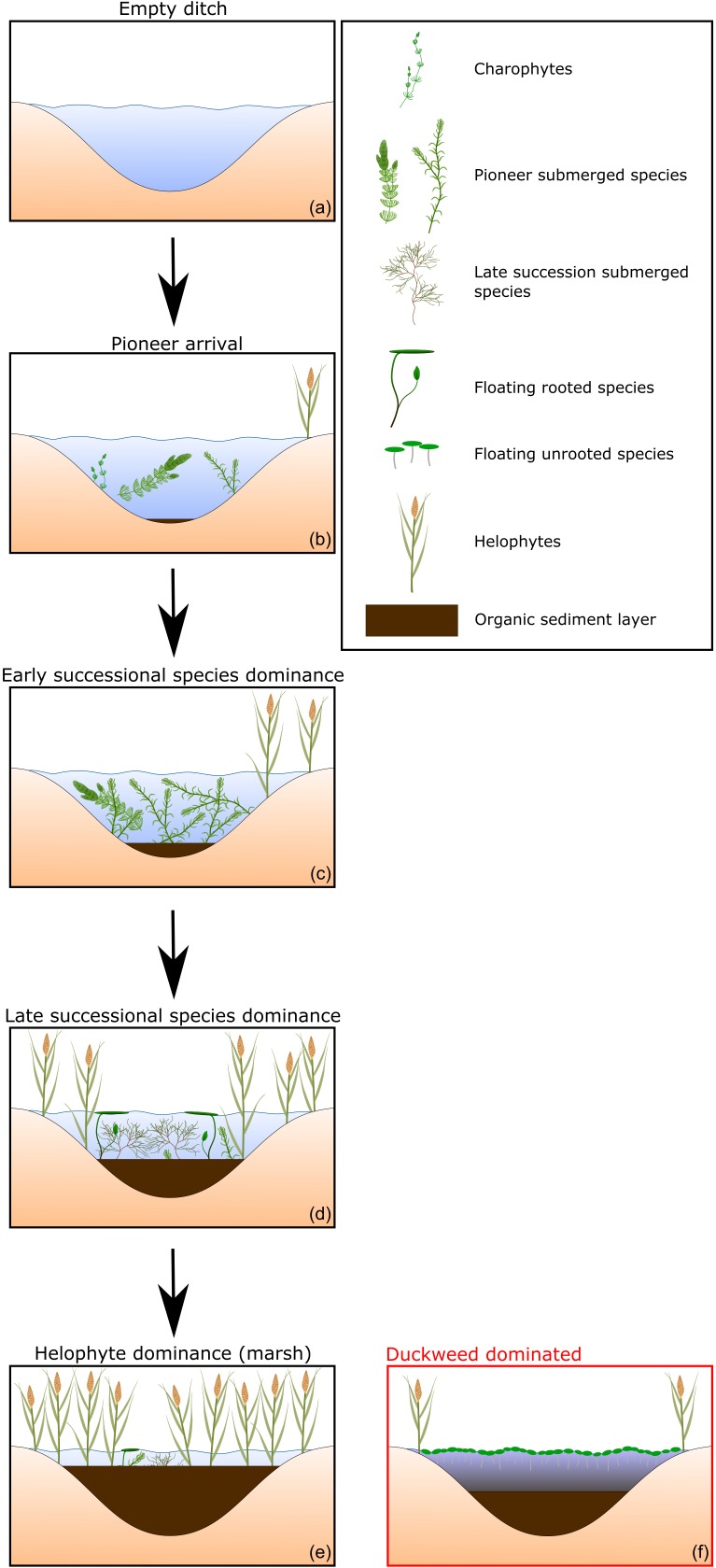
Schematic representation of the progression in the composition of functional vegetation types and organic sediment accumulation characteristic for vegetation succession in drainage ditches **(a–e)**. Under high nutrient load duckweeds may dominate hampering natural succession of the vegetation **(f)**.

Succession of aquatic vegetation in ditches is known to follow a general trajectory (Caspers and Heckman, 1981; Barendregt et al., 1992; Portielje and Roijackers, 1995; Lamers et al., 2002; Watson and Ormerod, 2004). After a short barren stage (**Figure 2a**), succession starts off with sparse patches of early successional submerged species, such as charophytes and vascular plants such as *Ceratophyllum demersum* and *Elodea nuttalli* (**Figure 2b**). Due the production and subsequent sedimentation of plant material the sediment will become covered by a thin layer of organic matter. The fastest growing species eventually become dominant. Due to a higher biomass production the organic sediment will builds up (**Figure 2c**). With increasing organic sediment layer thickness, the internal nutrient release will increase as well. This increase in nutrient status will favor different species groups, submerged groups such as *Potamogeton* sp. and rooted floating species such as *Nuphar lutea* (**Figure 2d**). Eventually, the organic sediment layer will build up to the point that helophytes are able to occupy the center of the ditch (**Figure 2e**). If left unmanaged a carr will remain. Under some conditions, succession deviates from this typical trajectory. Given high nutrient loading, the water surface may become entirely covered with duckweed (**Figure 2f**). Dominance by duckweed is believed to represent an alternative stable state which stabilizes itself via its impact on the aquatic environment. For example, high cover by duckweed results in anoxic conditions and a strongly reduced solar irradiance (Scheffer et al., 2003). This hampers the establishment of submerged and rooted floating plants (van Gerven et al., 2015) and as such prevents the progression of succession.

We defined functional types of vegetation known to be indicative for different successional stages (Barendregt et al., 1992; Lamers et al., 2002; Watson and Ormerod, 2004): charophytes, submerged pioneer vegetation, submerged late successional vegetation, rooted floating vegetation, unrooted floating vegetation (e.g., duckweeds) and helophyte vegetation (**Figure 2**). Through cluster analysis we grouped reaches according to successional stage properties (relative cover by vegetation functional types and sediment thickness). To select the most suitable cluster technique and the optimal number of clusters we compared *k*-means-, fuzzy-, self-organizing tree algorithm- and model based clustering approaches with the clValid package in R (Brock et al., 2011) using silhouette width and connectivity as the selection criteria. Based on functional type composition and sediment thickness, we ranked the clusters along the successional stage continuum as described above (**Figure 2**). All reaches were assigned to a cluster which represents their successional stage for further analyses.

### Successional Stages and Diversity

In this study, the two focal biodiversity indices are species richness (SR) and the species numbers equivalent of the Shannon diversity (*H*′). These two measures of diversity were chosen as they can be correctly partitioned into alpha and beta components in an additive fashion (Jost, 2007). *H*′ is calculated as the exponent of the Shannon-Wiener index and may be interpreted as a measure of the richness of abundant species in the community. For each of both indices, using the diversity partitioning framework of Jost (2007) and Jost et al. (2010), landscape diversity (γ) for each polder was partitioned into the average local diversity of ditch reaches (α- diversity) and a β-diversity component describing community dissimilarity among reaches. If different successional stages differ in community composition, then successional stage variation among ditch reaches will affect γ-diversity through the β-component. To evaluate the impact of such among-stage compositional differences on γ-diversity β-diversity was further partitioned into a component reflecting differences among communities within successional stages (β_within_), and a component reflecting community compositional differences among successional stages (β_between_). β_within_ was calculated as the average of β-diversity values calculated for each successional stage in each polder. β_between_ was calculated across successional stages within the polder. For species richness this was done using the list of species present in each stage (presence/absence data). For *H*′, we used the average abundances of species calculated across ditch reaches within successional stages instead.

To investigate whether compositional differences among successional stages arise from true species replacement patterns we partitioned the β_between_ component into two additional additive components, a component of ‘true species replacement’ (β_repl_) and a ‘richness difference’ component (β_rich_) using the approach proposed by Podani and Schmera (2011). For richness, the repl and rich partitions were calculated from a Jaccard-based multi-site β-diversity index (Ensing and Pither, 2015). To our knowledge, no such indices have been developed for abundance data. Instead, for the latter type of data we partitioned the total variance of a Ruzicka dissimilarity matrix following Legendre (2014).

We also tested for differences in community composition among all and between combinations of successional stages using distance-based Redundancy Analysis (dbRDA, Legendre and Anderson, 1999). Presence/absence data were analyzed using a Jaccard dissimilarity matrix among reaches within landscapes. Similarly, abundance data were analyzed using the Ruzicka dissimilarity index. dbRDAs were also performed on the species replacement (β_repl_) and richness difference (β_rich_) components of these indices (Legendre, 2014).

Typically, successional stages were represented by different numbers of reaches within landscapes. As differences in sample size may result in biased estimations of β-diversity components and incorrect significance values of RDA analyzes (Anderson and Walsh, 2013) we applied a random resampling procedure ensuring an equal representation of successional stages in each of these analyses. First, the stage with the least number of reaches in a polder was identified. Then β-diversity components were estimated and RDA analyses were performed on 1000 equally sized random draws of reaches from the different successional stages in the polder. Per polder, the resulting β-diversity values and RDA-associated *p*-values and test statistics were averaged across these permutations.

### Scenario Analyses Through Simulations: Evaluating the Effect of Successional Stage Heterogeneity on γ-Diversity

To test for the effect of removing successional stages from the landscape on γ-diversity we defined two main scenarios related to ditch management. The ‘Selective Management Scenario’ represents a management that gives priority to resetting succession in the late successional stages (e.g., through dredging). For this scenario we simulated seven sub scenarios. Together these sub-scenarios represent a gradient of increased management intensity along which the most advanced successional stages (late in the successional trajectory) are successively being removed. One extreme end of this gradient thus represents a situation where all stages are present whereas the other extreme pertains to a landscape that only contains reaches with stage 1 (earliest stage in the successional trajectory). The 2nd scenario, further referred to as the ‘No Management Scenario’ represents a situation in which management has stopped taking place and where the succession of the vegetation in each site progresses through the subsequent successional stages until it reaches the most advanced stage. Also for this scenario, we simulated seven sub scenarios. In contrast to the Selective Management Scenario the earliest stages are progressively removed from the landscape, starting from stage 1 and working forward (e.g., removing stages 1 and 2, stages 1 to 3, etc.) up to the point that a landscape consists solely of reaches with succession stage 7 (the latest in the successional trajectory).

Simulations were performed for each of the main scenarios separately. For each of the sub scenarios of a given main scenario, we assigned 12 ditches to each of 21 simulated landscapes. This was done by taking random draws of reaches from our vegetation dataset but respecting the constraints that define each of the sub-scenarios. This approach makes the explicit assumption of complete interchangeability of all reaches in the dataset, irrespective of polder identity. This implies that in our simulated landscape habitat suitability for species is determined by the successional stage alone. Furthermore, all species are deemed capable of reaching all the sites in the landscape (i.e., no dispersal limitation). For each landscape × sub-scenario combination, we partitioned γ into its α and β components and β into its richness and replacement components. For each of these end point variables, we studied its association with successional stage heterogeneity using linear regression of the form *y* = *a*^∗^*x*+*b* with y being the diversity end point variable, *x* being the successional stage heterogeneity of the sub scenario. This process was repeated 2000 times and we calculated average *p*-value and coefficients for the resulting 2000 regression lines. To study the diversity of individual successional stages we employed the same simulation strategy with each sub-scenario consisting of 12 randomly drawn ditch reaches of the same successional stage.

All analyses and simulations were performed in R version 3.3.2 using the *ggplot2* and *vegan* (Oksanen et al., 2015) packages and the custom code supplied by (Legendre, 2014).

## Results

### Identifying Successional Stages

**FIGURE 3:**
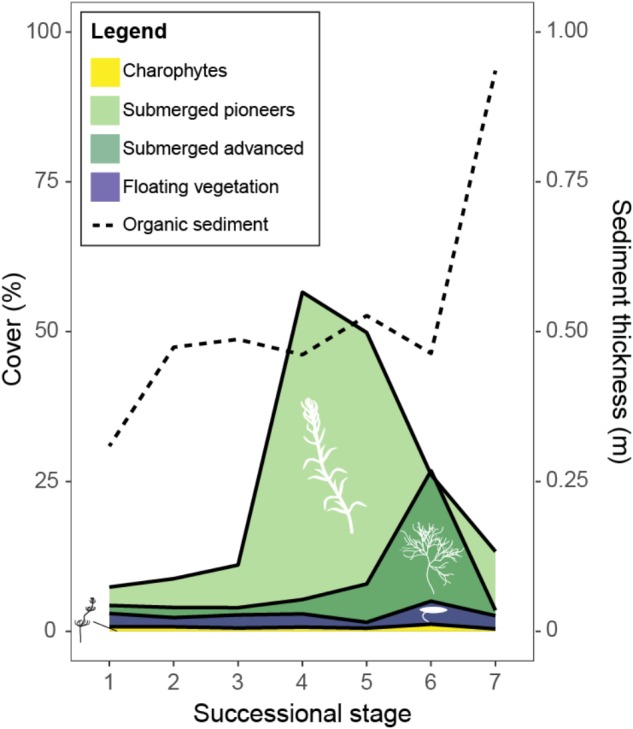
Progression of plant functional group cover (%) and organic sediment thickness (m) through the different identified successional stages (1–7). Averages are given of ditch reaches per identified successional stage. For data on relative cover for each of the species: see Supplementary Figure S1.

Fuzzy clustering yielded a solution with nine clusters and was found to provide the best fit (connectivity: 192.8; silhouette width: 0.241). Based on functional type composition of the vegetation and sediment thickness, seven of these clusters could be identified as successional stages and ranked along the succession gradient as described in **Figures 2**, **3** and Supplementary Figure S1. Stage 1 was characterized primarily by low abundance of submerged vegetation and a thin organic sediment layer. Compared to Stage 1, Stages 2 and 3 had a thicker sediment layer and showed an increased abundance of submerged pioneering species. Stages 2 and 3 were very similar and differed mainly in cover by floating unrooted vegetation (Supplementary Figure S1). Compared to the previous stages, Stage 4 showed a strong increase in the cover by submerged pioneers. Similarly to Stage 4, Stage 5 contained a relatively large population of submerged pioneers but also had a larger share of late successional submerged plants. In Stage 6 late successional submerged species became dominant, floating rooted plants increased while early pioneers decreased in abundance. Ditch reaches belonging to Stage 7 were characterized by a relatively thick layer of organic sediment and an overall decline of true aquatic vegetation. Very advanced successional stages with a predominance of helophytes in the middle of the ditch did not occur in our dataset, most probably as the result of current management. Generally, helophytes were growing along the ditch margins in all successional stages and proved a poor indicator of successional stage (not shown in **Figure 3**). One cluster corresponded best with a duckweed dominated alternative stable state and could as such not be categorized as a successional stage. Another cluster was characterized by a relatively thick organic sediment layer and overall low vegetation cover, possibly caused by high local sedimentation rates or a combination of frequent vegetation removal with lack of dredging. This stage is also rather atypical and was not categorized as a successional stage either.

### Diversity in Successional Stages: Partitioning Results

**FIGURE 4:**
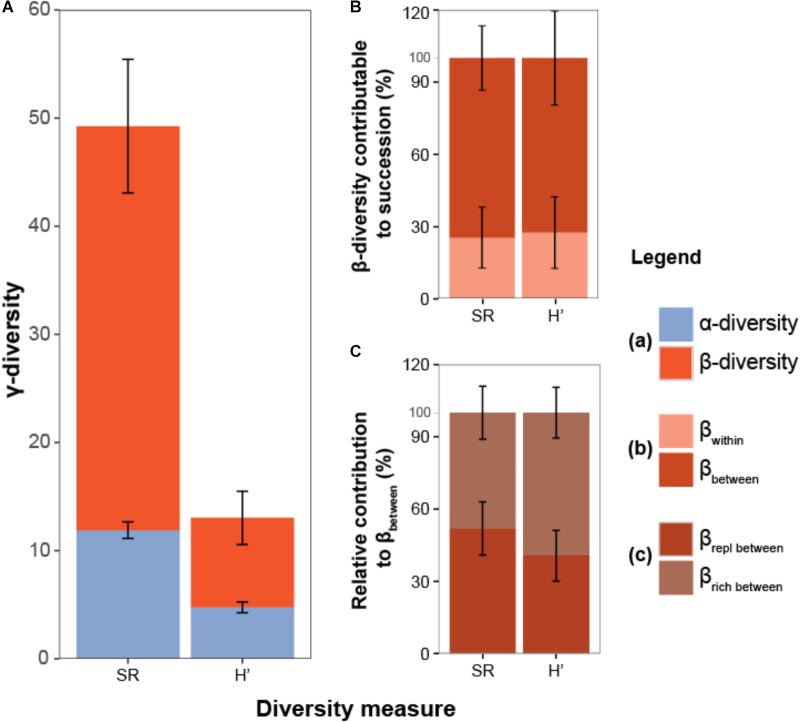
**(A)** Total observed diversity (γ) of ditch vegetation within polders partitioned into components of: (1) mean local diversity of ditch reaches (α), (2) difference in community composition between reaches within polder landscapes (β). **(B)** β-diversity is further divided into a component attributable to compositional variation among reaches belonging to the same successional stages (β_within_) and variation among reaches from different successional stages (β_between_). **(C)** Relative contribution of species replacement (β_repl_) and richness differences (β_rich_) (Legendre, 2014). Partitioning results are shown for both species richness (SR) and the exponent of the Shannon-Wiener index (*H*′). Error bars reflect variation among polders and equal twice the standard error around the mean.

The partitioning of γ-diversity into its α and β components (**Figure 4A**) revealed that only a small part of the landscape-wide diversity (γ) could be attributed to the local diversity (α) of ditch reaches (SR = 11.9, *H*′ = 4.8). In contrast, the contribution of the β component was much higher (SR = 75%, *H*′ = 60%), indicating large differences in community composition among ditch reaches within polders. A large part of the β-diversity could be attributed to differences between successional stages (β_between_: SR = 80.4%, *H*′ = 77.3%, **Figure 4B**) and about half of this β-diversity (β_between_) represented true replacement of species (SR: 51.7%, *H*′ = 40.4%; **Figure 4C**).

The dbRDA-analyses showed significant differences in the community composition of successional stages (Jaccard: mean *R*_adj_^2^ = 5.2%^∗∗∗^, Ruzicka: mean *R*_adj_^2^ = 25.6%^∗∗∗^). Species replacement was significantly associated with differences between successional stages (Jaccard: *R*_adj_^2^ = 4.4%

### Effects of Management Scenario’s on Diversity

**FIGURE 5:**
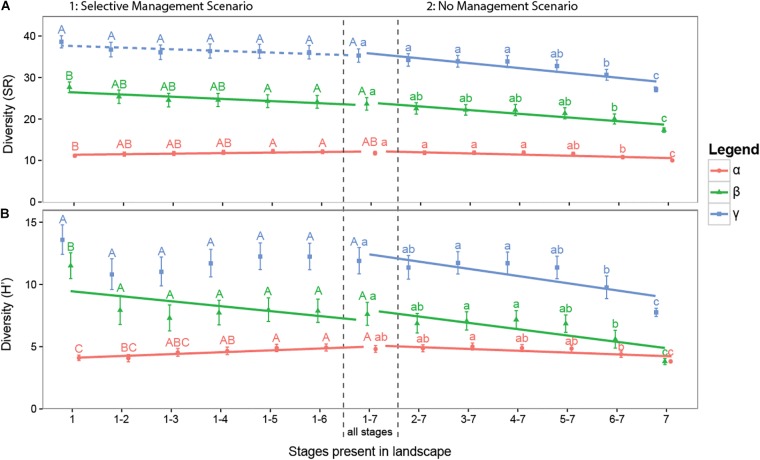
Simulation results of scenario analyses showing the effects of decreasing landscape-wide successional stage heterogeneity on the landscape diversity (γ) and its partitions (α, β) for both species richness **(A)** and true Shannon diversity *H*′ **(B)**. On the left hand side the Selective Management scenario is shown, where late successional stages are progressively removed from the landscape. On the right hand side the No Management scenario is shown, where early successional stages are progressively removed from the landscape. Error bars show twice the standard error around the landscape-wide mean diversity values based on 21 simulated landscapes with 12 ditch reaches per landscape. Letters indicating pairwise significance between successional group means. Lines show significant trends (*p* < 0.05) along the gradient of management based on a linear model and dashed lines show marginally significant trends (*p* < 0.10).

With scenario analyses we assessed potential changes in the γ-diversity through management associated reductions in landscape-wide successional stage heterogeneity. Landscapes containing all seven successional stages had a mean γ-diversity of 35 species (**Figure 5**). In the Selective Management Scenario, where late successional stages were progressively removed from the landscape, we did not find a significant change in the γ-diversity (LM: intercept = 35.21, slope = 0.40, *p* = 0.0502, *R*_adj_^2^ = 6.8%). In contrast, the gradual disappearance from the landscape of early successional stages in the No Management Scenario resulted in a significant reduction of the γ-diversity with a total loss of on average seven species or about 20% of the species in a landscape (LM: intercept = 36.36, slope = -1.17, *p* < 0.001, *R*_adj_^2^ = 36.5%). Although this trend was associated with a reduced α-diversity, it seemed to be mainly caused by a reduction in β-diversity (**Figure 5B** and Supplementary Table S3).

To further elucidate the diversity decline observed for the No Management Scenario, we partitioned the β-diversity between successional stages (β_between_) into its replacement (β_repl_) and richness difference (β_rich_) components. Supplementary Figure S4 and Supplementary Table S4 shows that the decline in β-diversity of the reaches in the landscape (β_between_) was entirely driven by a decline in β_repl_, i.e., complementarity among successional stages within the landscape (LM: intercept = 0.39, slope = -0.045, *p* < 0.001, *R*_adj_^2^ = 33.7%) and not by the loss of richness differences among successional stages, β_rich_ (*p* = 0.401). Similar analysis on species abundance, giving greater weight to highly abundant or dominant species, showed no effect on the total β_between_ (*p* = 0.124). Results of species richness and Shannon diversity were similar (Supplementary Table S4).

By running sub-scenarios with landscapes consisting of only one successional stage we were able to assess how successional stages differed from each other in terms of their diversity. A landscape consisting of just the first successional stage is very divers with, respectively, 38 and 14 species for SR and *H*′ (**Figure 5**). Landscapes consisting of the later successional stages are less divers with a landscape consisting of stage 7 alone consisting of only, respectively, 27 and 7 species for SR and *H*′. The local diversity (α) of the different stages is relatively similar, ranging from 10 to 11 species for SR and 4 to 5 for *H*′. The β-diversity of the different stages varied widely (SR: from 29 to 19 species and *H*′: 12 to 4 species) with the earliest stage showing the greatest within-stage heterogeneity among ditch reaches.

## Discussion

Our diversity partitioning analyses show that β-diversity forms a very important component of the γ-diversity of macrophytes in ditch networks of polder systems. These results are in agreement with several other studies that have shown large differences in community composition among sites for a variety of organism groups in agricultural ditches, including macroinvertebrates, diatoms and helophytes and aquatic vegetation (Leng et al., 2010; van Zuidam and Peeters, 2013; Goldenberg Vilar et al., 2014; Clarke, 2015). New to our analyses is that more than 70% of this β-diversity seems to arise from differences in successional stages of ditch vegetation and that at least 40% of this β-diversity stems from complementarities in species composition between successional stages. Our results thus clearly demonstrate that heterogeneity in successional stages contribute strongly to the regional diversity of macrophytes in the ditch network of a landscape.

The importance of landscape heterogeneity for biodiversity has been shown in a variety of biomes such as boreal forests, grasslands and rivers (respectively, Niemala et al., 1996; Richards et al., 1999; Ward et al., 2002). Here we showed similar results for aquatic plants in agricultural drainage ditches within polder landscapes, illustrating the importance of maintaining landscape heterogeneity. The large contribution of β-diversity to γ-diversity observed in our study emphasizes the necessity for a focus of management on diversity at the landscape scale rather than at the scale of local ditch reaches. Given that differences among successional stages form an important part of the landscape heterogeneity influencing the β-diversity in our polder landscapes, the creation and maintenance of successional heterogeneity may be an important way through which management may be able to enhance and sustain landscape wide biodiversity. In the absence of any management or natural disturbances, heterogeneity among communities of different sites will gradually be reduced as succession progresses (Vandvik et al., 2005). The landscape would increasingly be dominated by a limited number of late successional stages. As the ‘No Management Scenario’ of our simulations illustrate, this is expected to result in a gradual decrease of β- and γ-diversity through time, as the early successional stages are lost from the landscape.

Currently, management in the agricultural ditch networks of Netherlands is almost entirely focused on the maintenance of their hydrological function and largely involves regular dredging, vegetation removal and bank reshaping (Twisk et al., 2003). Given that these measures involve a reset of succession, they contribute to the rejuvenation of vegetation (Wade, 1993) and may as such also contribute to the maintenance of successional stage heterogeneity and γ-diversity. However, the impact of these measures on the latter variables will also depend on the frequency and the scale of their application. The frequent and simultaneous application of these measures over large spatial areas will inevitably result in the disappearance of late successional stages. According to our diversity partitioning results this would be expected to result in a reduced β- and γ-diversity as well. However, such expectation is poorly supported by our simulation results which suggest that a predominance of young successional stages would be of little consequence to γ-diversity. Indeed, in these simulations, the progressive loss of later successional stages resulted in a minor reduction of α-diversity, but this was entirely compensated by an increase in β-diversity. Across the entire dataset, β-diversity of early successional stages was relatively high compared to late successional stages (Supplementary Figure S5). The high compositional variability among communities of early successional stages likely reflects a large impact of stochasticity during community assembly. Compared to late successional stages, interspecific interactions in young habitat patches are still weak and the composition of pioneering communities is likely more determined by coincidental dispersal and colonization events than by the outcome of competition (Tilman, 1994).

When interpreting the simulation results it is, however, important to note that diversity indices were calculated based on random draws from the entire dataset. Communities of ditch reaches were thus regarded to be freely interchangeable among polders. This implicitly assumes absence of any dispersal limitation and ignores historic trajectories of metacommunities in individual polders. This may have led to an underestimation of the negative effects of late successional stage loss. By removing late successional stages, their function as source of populations recolonizing the earlier stages may be compromised. The diversity of early successional stages in the absence of late successional stages may therefore have been overestimated in our simulations. In reality the reassembly of communities after the reset of succession may largely depend on colonization, which may take 3–4 years to recover full vegetation diversity (Milsom et al., 2004). Colonization by dispersing propagules from proximate source communities is also likely to be more influential than from more distant ones (Brederveld et al., 2011; Van Dijk et al., 2014). Furthermore, the remaining seed bank can also play an important role in community reestablishment (Sarneel et al., 2014; Van Leeuwen et al., 2014), although it is unknown to what extent dredging activities reduce local community resilience by removing large parts of this reservoir. If management resets succession in large parts of the landscape simultaneously, recolonization of empty sites may be severely impeded by dispersal limitation reducing overall β- and γ-diversity of the landscape. Although the contribution of early successional stages to γ-diversity may potentially be disproportionally high, the role of nearby mid and late successional stages as source communities of dispersal propagules should not be ignored in real plant metacommunities.

Conceptually, CRM is likely the type of management scheme that best guarantees the presence of a range of successional stages in the landscape. However, much depends on the specific parameters of management, namely the delay and frequency of visit by management (Hinsch and Poethke, 2007). The delay is defined as the time that each site is left to develop naturally before it is revisited (Morris, 2000). Frequency of visits by management to the landscape is defined as the proportion of sites being managed during each visit. More frequent visits lead to a lower proportion of sites being managed per visit, but creating a higher variation in successional age within the landscape (Hinsch and Poethke, 2007). A low delay (the duration of a few years) may entirely exclude some late successional stages from the landscape and therefore eliminate potential sources for recolonization of recently managed ditch reaches. In contrast, high delays (decades) may result in a predominance of late successional stages that may interfere with the hydrological function of the ditches and contribute less to γ-diversity. When a large number of ditch reaches are visited at once (low frequency), a landscape will likely consisting of large, homogenous blocks with relatively low successional stage diversity across the landscape. Low frequency management is typically found in the currently employed, large scale dredging operations where large swaths of the landscape are managed at the same time. We advocate that application of CRM management schemes with more frequent visits and possibly also higher delays would lead to more heterogeneous mosaic-like landscape patterns (see Niemala et al., 1996 for an example in boreal forests). Such mosaic may be further elaborated through a spatial planning that is focused on minimizing distance between recently managed and other reaches as to facilitate dispersal and recolonization (Watson et al., 2012; Van Dijk et al., 2014).

Although the potential advantages of CRM for biodiversity conservation in agricultural ditch networks are obvious, it is difficult to make generalizations about what should be the optimal CRM scheme. Ideally, CRM schemes are based on knowledge about the development rates of the different successional stages. Succession rates appear to be highly variable as the time needed for succession to turn an empty ditch into a carr may range between 10 and 27 years (Bakker et al., 1994). Succession rates in aquatic systems are highly context specific and depend on several factors such as sedimentation rates, flow velocity (Sousa, 1979), sediment characteristics, intensity of land use and associated nutrient loading (Carpenter, 1981). Obviously, more productive systems with high rates of sediment loading will require CRM schemes with shorter delays than less productive systems. Ideally, CRM schemes are adapted and improved based on information acquired through the monitoring of succession in parts of the ditch network that have been managed, a form of adaptive management (Haney and Power, 1996). Using social-benefit analyses (e.g., Fliervoet et al., 2013) the benefits to farmers (reduced management effort and cost), water quantity managers (maintained drainage capacity) and nature water quality organizations (increased ecological diversity) may be quantified in detail to arrive at an optimal management plan for a specific landscape.

It’s important to note that the ditch networks considered in our study have historically been associated with intensive land use. They are biotically impoverished in comparison to ditch networks in areas with less intensive agriculture (Leng et al., 2010). High nutrient loading is likely to enhance the intensity of interspecific competition especially in the later successional stages, leading to monotonous vegetation (van Zuidam and Peeters, 2013). This may have contributed to the relatively high degree of homogeneity of these stages in the landscape. Furthermore, conclusions of this study only pertain to the successional stages that were encountered in our field study. Current management practices in the Dutch agricultural polder landscape prevent ditches from containing successional stages that are older and more developed that the ones observed in our studies (e.g., alder carrs, floating fens). Our analyses and simulations do not take into account the potential contribution of such successional stages to β- and γ-diversity. Although such stages likely interfere with the drainage function of the ditch network, they would potentially have a unique and important contribution to regional diversity. Hence it would be interesting to also evaluate such stages in future work and consider how they can be maintained in the landscape.

The same case can be made for other groups of organisms that make their home in the ditch systems. Despite the important structural and functional role of macrophytes in the aquatic habitat, conservation management in ditch networks should also aim at supporting biodiversity of other organism groups. Extrapolation of our conclusions regarding the contribution of successional stages to macrophyte biodiversity should therefore be done with prudence. Although the diversity of macrophytes may be an important determinant for the diversity of some associated groups (Whatley et al., 2014b), such association is not unequivocally strong for all aquatic groups. Producers are shown to be more strongly driven by patterns of replacement than higher trophic groups (Soininen et al., 2018). Therefore, studies on the impact of succession on the β- and γ-diversity of those other groups and how these can be adequately managed is vital. Our data driven approach may be easily applied to other species groups to assess the potential efficacy of a CRM scheme. Herein lays the power of our approach, all it requires is data on species composition of different sites in a landscape and a conceptual notion of successional trajectories. This type of data is generally gathered by nature- and water managers in their standard monitoring schemes. These data may serve as an ideal first step in evaluating the inherent requirements (differences in, and replacement of species between successional stages) for useful application of CRM, before extensive management activities are carried out that may be hit-or-miss. Further development of a specific management scheme could benefit from specific deterministic models (see Perona et al., 2009 for CRM in floodplains and Larocque et al., 2013 for an overview of similar models in forests).

## Conclusion

We have shown the importance of succession as a driving force in agricultural drainage ditches, non-natural aquatic habitats that nonetheless can contribute to the biodiversity of the landscape (Davies et al., 2008; Verdonschot et al., 2011). Promoting successional heterogeneity is possible through cyclic rejuvenation of parts of the landscape through management. As management of ditch systems is needed to preserve hydrological functioning (Herzon and Helenius, 2008), the change to a system-specific, spatially explicit management scheme employing the principles of CRM can be both viable and cost-effective. This combination of drainage and ecological function fits well within the context of reconciliation ecology (Rosenzweig, 2003), allowing for coexistence of relatively high levels of biodiversity in a seemingly unhospitable landscape of anthropogenic agricultural activity.

## Author Contributions

ST, MV, and SD conceived the conceptual idea for the study. ST and SD designed the study and ST carried out the field work. ST carried out the data analysis with help from SD and MV. The first draft of the manuscript was written by ST, MV, EB, and SD with all authors contributing substantially to revisions.

## Conflict of Interest Statement

The authors declare that the research was conducted in the absence of any commercial or financial relationships that could be construed as a potential conflict of interest. The handling Editor is currently co-organizing a Research Topic with one of the authors EB, and confirms the absence of any other collaboration.

## References

[B1] Anderson M. J. Walsh D. C. I. (2013). PERMANOVA, ANOSIM, and the mantel test in the face of heterogeneous dispersions: what null hypothesis are you testing? *Ecol. Monogr.* 83 557–574. 10.1890/12-2010.1

[B2] Armitage P. D. Szoszkiewicz K. Blackburn J. H. Nesbitt I. (2003). Ditch communities: a major contributor to floodplain biodiversity. *Aquat. Conserv.* 13 165–185. 10.1002/aqc.549

[B3] Bakker S. A. Berg N. J. van den Speleers B. R. (1994). Vegetation transitions of floating wetlands in a complex of turbaries between 1937 and 1989 as determined from aerial photographs with GIS. *Vegetatio* 114 161–167.

[B4] Baptist M. J. Penning W. E. Duel H. Smits A. J. M. Geerling G. W. van der Lee G. E. M. (2004). Assessment of the effects of cyclic floodplain rejuvenation on flood levels and biodiversity along the rhine river. *River Res. Appl.* 20 285–297. 10.1002/rra.778

[B5] Barendregt A. Stam S. M. E. Wassen M. J. (1992). Restoration of fen ecosystems in the Vecht river plain: cost-benefit analysis of hydrological alternatives. *Hydrobiologia* 233 247–258. 10.1007/BF00016113

[B6] Baselga A. (2010). Partitioning the turnover and nestedness components of beta diversity. *Glob. Ecol. Biogeogr.* 19 134–143. 10.1111/j.1466-8238.2009.00490.x

[B7] Brederveld R. J. Jähnig S. C. Lorenz A. W. Brunzel S. Soons M. B. (2011). Dispersal as a limiting factor in the colonization of restored mountain streams by plants and macroinvertebrates. *J. Appl. Ecol.* 48 1241–1250. 10.1111/j.1365-2664.2011.02026.x

[B8] Brock G. Pihur V. Datta S. Datta S. (2011). *Clvalid, an R Package for Cluster Validation.* Available at: http://cran.us.r-project.org/web/packages/

[B9] Carpenter S. R. (1981). Submersed vegetation: an internal factor in lake ecosystem succession. *Am. Nat.* 118 372–383. 10.1086/283829

[B10] Caspers H. Heckman C. W. (1981). Ecology of orchard drainage ditches along the freshwater section of the Elbe Estuary. *Arch. Hydrobiol.* 43 347–486.

[B11] Catry I. Marcelino J. Franco A. M. A. Moreira F. (2017). Landscape determinants of european roller foraging habitat: implications for the definition of agri-environmental measures for species conservation. *Biodivers. Conserv.* 26 553–566. 10.1007/s10531-016-1241-1244

[B12] Clarke S. J. (2015). Conserving freshwater biodiversity: the value, status and management of high quality ditch systems. *J. Nat. Conserv.* 24 93–100. 10.1016/j.jnc.2014.10.003

[B13] Davies B. Biggs J. Williams P. Whitfield M. Nicolet P. Sear D. (2008). Comparative biodiversity of aquatic habitats in the european agricultural landscape. *Agric. Ecosyst. Environ.* 125 1–8. 10.1016/j.agee.2007.10.006

[B14] Dornelas M. Gotelli N. J. McGill B. Shimadzu H. Moyes F. Sievers C. (2014). Assemblage time series reveal biodiversity change but not systematic loss. *Science* 344 296–300. 10.1126/science.1248484 24744374

[B15] Ensing D. J. Pither J. (2015). A novel multiple-site extension to pairwise partitioned taxonomic beta diversity. *Ecol. Complex.* 21 62–69. 10.1016/j.ecocom.2014.11.008

[B16] Fliervoet J. M. Van den Born R. J. G. Smits A. J. M. Knippenberg L. (2013). Combining safety and nature: a multi-stakeholder perspective on integrated floodplain management. *J. Environ. Manage.* 128 1033–1042. 10.1016/j.jenvman.2013.06.023 23911983

[B17] Foley J. A. Defries R. Asner G. P. Barford C. Bonan G. Carpenter S. R. (2005). Global consequences of land use. *Science* 309 570–574. 10.1126/science.1111772 16040698

[B18] Goldenberg Vilar A. Van Dam H. Van Loon E. E. Vonk J. A. Van Der Geest H. G. Admiraal W. (2014). Eutrophication decreases distance decay of similarity in diatom communities. *Freshw. Biol.* 59 1522–1531. 10.1111/fwb.12363

[B19] Haney A. Power R. L. (1996). Adaptive management for sound ecosystem management. *Environ. Manage.* 20 879–886. 10.1007/BF01205968 8895410

[B20] Herzon I. Helenius J. (2008). Agricultural drainage ditches, their biological importance and functioning. *Biol. Conserv.* 141 1171–1183. 10.1016/j.biocon.2008.03.005

[B21] Hill R. P. Chadd N. Morris M. J. Swaine P. J. Wood J. D. (2016). Aquatic macroinvertebrate biodiversity associated with artificial agricultural drainage ditches. *Hydrobiologia* 10.1007/s10750-016-2757-z

[B22] Hinsch M. Poethke H. (2007). Consequences of cyclic vegetation management for arthropod survival: simulation experiments. *Basic Appl. Ecol.* 8 321–331. 10.1016/j.baae.2006.09.011

[B23] Jost L. (2007). Partitioning diversity into independent alpha and beta components. *Ecology* 88 2427–2439. 10.1890/06-1736.1 18027744

[B24] Jost L. Devries P. Walla T. Greeney H. Chao A. Ricotta C. (2010). Partitioning diversity for conservation analyses. *Divers. Distrib.* 16 65–76. 10.1111/j.1472-4642.2009.00626.x

[B25] Lamers L. P. M. Smolders A. J. P. Roelofs J. G. M. (2002). The restoration of fens in the Netherlands. *Hydrobiologia* 478 107–130. 10.1023/A:1021022529475

[B26] Larocque G. R. Luckai N. Adhikary S. N. Groot A. Bell F. W. Sharma M. (2013). Competition theory — science and application in mixed forest stands: review of experimental and modelling methods and suggestions for future research. *Environ. Rev.* 21 71–84. 10.1139/er-2012-2033

[B27] Legendre P. (2014). Interpreting the replacement and richness difference components of beta diversity. *Glob. Ecol. Biogeogr.* 23 1324–1334. 10.1111/geb.12207

[B28] Legendre P. Anderson M. J. (1999). Distace-based redundancy analysis: testing multispecies responses in multifactorial experiments. *Ecol. Monogr.* 69 1–24. 10.1890/0012-9615(1999)069[0001:DBRATM]2.0.CO;2

[B29] Leng X. Musters C. J. M. de Snoo G. R. (2010). Spatiotemporal variation of plant diversity on ditch banks under different management regimes. *Basic Appl. Ecol.* 12 38–46. 10.1016/j.baae.2010.10.005

[B30] McGill B. J. Dornelas M. Gotelli N. J. Magurran A. E. (2015). Fifteen forms of biodiversity trend in the anthropocene. *Trends Ecol. Evol.* 30 104–113. 10.1016/j.tree.2014.11.006 25542312

[B31] Milsom T. P. Sherwood A. J. Rose S. C. Town S. J. Runham S. R. (2004). Dynamics and management of plant communities in ditches bordering arable fenland in eastern England. *Agric. Ecosyst. Environ.* 103 85–99. 10.1016/j.agee.2003.10.012

[B32] Morris M. G. (2000). The effects of structure and its dynamics on the ecology and conservation of arthropods in British grasslands. *Biol. Conserv.* 95 129–142. 10.1016/S0006-3207(00)00028-28

[B33] Niemala J. Haila Y. Punttila P. Niemela J. (1996). The importance of small-scale heterogeneity in boreal forests: variation in diversity in forest-floor invertebrates across the succession gradient. *Ecography* 19 352–368. 10.1111/j.1600-0587.1996.tb01264.x

[B34] Odum E. P. (1969). The strategy of ecosystem development. *Science* 164 262–270. 10.1126/science.164.3877.262 5776636

[B35] Oksanen J. Blanchet F. G. Kindt R. Legendre P. Minchin P. R. O’Hara R. B. (2015). *vegan: Community Ecology Package. R Package Version 2.3–1.*

[B36] Perona P. Camporeale C. Perucca E. Savina M. Molnar P. Burlando P. (2009). Modelling river and riparian vegetation interactions and related importance for sustainable ecosystem management. *Aquat. Sci.* 71 266–278. 10.1007/s00027-009-9215-9211

[B37] Podani J. Schmera D. (2011). A new conceptual and methodological framework for exploring and explaining pattern in presence - absence data. *Oikos* 120 1625–1638. 10.1111/j.1600-0706.2011.19451.x

[B38] Portielje R. Roijackers R. M. M. (1995). Primary succession of aquatic macrophytes in experimental ditches in relation to nutrient input. *Aquat. Bot.* 50 127–140. 10.1016/0304-3770(94)00439-S

[B39] Power A. G. (2010). Ecosystem services and agriculture: tradeoffs and synergies. *Philos. Trans. R. Soc. Lond. B Biol. Sci.* 365 2959–2971. 10.1098/rstb.2010.0143 20713396 PMC2935121

[B40] Richards S. A. Possingham H. P. Tizard J. (1999). Optimal fire management for maintaining community diversity. *Ecol. Appl.* 9 880–892. 10.1890/1051-0761(1999)009[0880:OFMFMC]2.0.CO;2

[B41] Rosenzweig M. L. (2003). Reconciliation ecology and the future of species diversity. *Oryx* 37 194–205. 10.1017/S0030605303000371

[B42] Sarneel J. M. Janssen R. H. Rip W. J. Bender I. M. A. Bakker E. S. (2014). Windows of opportunity for germination of riparian species after restoring water level fluctuations: a field experiment with controlled seed banks. *J. Appl. Ecol.* 51 1006–1014. 10.1111/1365-2664.12288

[B43] Scheffer M. Szabo S. Gragnani A. Van Nes E. H. Rinaldi S. Kautsky N. (2003). Floating plant dominance as a stable state. *Proc. Natl. Acad. Sci. U.S.A.* 100 4040–4045. 10.1073/pnas.0737918100 12634429 PMC153044

[B44] Smart S. M. Thompson K. Marrs R. H. Le Duc M. G. Maskell L. C. Firbank L. G. (2006). Biotic homogenization and changes in species diversity across human-modified ecosystems. *Proc. R. Soc. B Biol. Sci. Soc.* 273 2659–2665. 10.1098/rspb.2006.3630 17002952 PMC1635461

[B45] Soininen J. Heino J. Wang J. (2018). A meta-analysis of nestedness and turnover components of beta diversity across organisms and ecosystems. *Glob. Ecol. Biogeogr.* 27 96–109. 10.1111/geb.12660

[B46] Sousa W. P. (1979). Experimental investigations of disturbance and ecological succession in a rocky intertidal algal community. *Ecol. Monogr.* 49 227–254. 10.2307/1942484

[B47] Sousa W. P. (1984). The role of disturbance in natural communities. *Annu. Rev. Ecol. Syst.* 15 353–391. 10.1146/annurev.es.15.110184.002033

[B48] Tansley A. G. (1946). *Introduction To Plant Ecology.* London: Allen & Unwin.

[B49] Tilman D. (1994). Competition and biodiversity in spatially structured habitats. *Ecology* 75 2–16. 10.2307/1939377

[B50] Tockner K. Malard F. Ward J. V. (2000). An extension of the flood pulse concept. *Hydrol. Process.* 14 2861–2883. 10.1002/1099-1085(200011/12)14:16/17<2861::AID-HYP124>3.0.CO;2-F

[B51] Twisk W. Noordervliet M. A. W. Ter Keurs W. J. (2003). The nature value of the ditch vegetation in peat areas in relation to farm management. *Aquat. Ecol.* 37 191–209. 10.1023/A:1023944028022

[B52] van der Plas F. Manning P. Soliveres S. Allan E. Scherer-lorenzen M. Verheyen K. (2016). Biotic homogenization can decrease landscape-scale forest multifunctionality. *Proc. Natl. Acad. Sci. U.S.A.* 113 3557–3562. 10.1073/pnas.1605668113 26979952 PMC4822601

[B53] Van Dijk W. F. A. Van Ruijven J. Berendse F. de Snoo G. R. (2014). The effectiveness of ditch banks as dispersal corridor for plants in agricultural landscapes depends on species’ dispersal traits. *Biol. Conserv.* 171 91–98. 10.1016/j.biocon.2014.01.006

[B54] Vandvik V. Heegaard E. Måren I. E. Aarrestad P. A. (2005). Managing heterogeneity: the importance of grazing and environmental variation on post-fire succession in heathlands. *J. Appl. Ecol.* 42 139–149. 10.1111/j.1365-2664.2005.00982.x

[B55] van Gerven L. P. A. de Klein J. J. M. Gerla D. J. Kooi B. W. Kuiper J. J. Mooij W. M. (2015). Competition for light and nutrients in layered communities of aquatic plants. *Am. Nat.* 186 72–83. 10.1086/681620 26098340

[B56] Van Leeuwen C. H. A. Sarneel J. M. van Paassen J. Rip W. J. Bakker E. S. (2014). Hydrology, shore morphology and species traits affect seed dispersal, germination and community assembly in shoreline plant communities. *J. Ecol.* 102 998–1007. 10.1111/1365-2745.12250

[B57] van Strien A. J. van der Burg T. Rip W. J. Strucker R. C. W. (1991). Effects of mechanical ditch management on the vegetation of ditch banks in dutch peat areas. *J. Appl. Ecol.* 28 501–513. 10.2307/2404564

[B58] van Zuidam J. P. Peeters E. T. (2013). Occurrence of macrophyte monocultures in drainage ditches relates to phosphorus in both sediment and water. *Springerplus* 2:564. 10.1186/2193-1801-2-564 24255858 PMC3825067

[B59] Verdonschot R. C. M. Keizer-Vlek H. E. Verdonschot P. F. M. (2011). Biodiversity value of agricultural drainage ditches: a comparative analysis of the aquatic invertebrate fauna of ditches and small lakes. *Aquat. Conserv.* 21 715–727. 10.1002/aqc.1220

[B60] Wade P. M. (1993). The influence of vegetation pre-dredging on the post-dredging community. *J. Aquat. Plant Manag.* 31 141–144.

[B61] Walker L. R. Zarin D. J. Fetcher N. Myster R. W. Johnson A. H. (1996). Ecosystem development and plant succession on landslides in the caribbean. *Biotropica* 28 566–576. 10.2307/2389097

[B62] Ward J. V. Tockner K. Arscott D. B. Claret C. (2002). Riverine landscape diversity. *Freshw. Biol.* 47 517–539. 10.1046/j.1365-2427.2002.00893.x

[B63] Watson A. M. Ormerod S. J. (2004). The distribution of three uncommon freshwater gastropods in the drainage ditches of British grazing marshes. *Biol. Conserv.* 118 455–466. 10.1016/j.biocon.2003.09.021

[B64] Watson S. J. Taylor R. S. Nimmo D. G. Kelly L. T. Clarke M. F. Bennett A. F. (2012). The influence of unburnt patches and distance from refuges on post-fire bird communities. *Anim. Conserv.* 15 499–507. 10.1111/j.1469-1795.2012.00542.x

[B65] Whatley M. H. van Loon E. E. van Dam H. Vonk J. A. van der Geest H. G. Admiraal W. (2014a). Macrophyte loss drives decadal change in benthic invertebrates in peatland drainage ditches. *Freshw. Biol.* 59 114–126. 10.1111/fwb.12252

[B66] Whatley M. H. van Loon E. E. Vonk J. A. van der Geest H. G. Admiraal W. (2014b). The role of emergent vegetation in structuring aquatic insect communities in peatland drainage ditches. *Aquat. Ecol.* 48 267–283. 10.1007/s10452-014-9482-9483

[B67] Whittaker R. H. (1960). Vegetation of the siskiyou mountains, oregon and California. *Ecol. Monogr.* 30 279–338. 10.2307/1943563

[B68] Whittaker R. J. (1970). *Communities and Ecosystems* 1st Edn New York, NY: Macmillan.

