# Supplementary material for: Managing Successional Stage Heterogeneity to Maximize Landscape-Wide Biodiversity of Aquatic Vegetation in Ditch Networks
Source: Front Plant Sci. 2018 Jul 16;9:1013. doi: 10.3389/fpls.2018.01013 (PMC6055429; doi:10.3389/fpls.2018.01013)
Supplement: Supplementary file 1 [file Data_Sheet_1.docx]

# Managing on successional stage heterogeneity to maximize landscape-wide biodiversity of aquatic vegetation

# Authors:

Sven Teurlincx^*^, Michiel J.J.M. Verhofstad, Elisabeth S. Bakker, Steven A.J. Declerck

*Corresponding author: S. Teurlincx, e-mail: [s.teurlincx@nioo.knaw.nl](mailto:s.teurlincx@nioo.knaw.nl), postal address: Droevendaalsesteeg 10, 6708 PB Wageningen, Gelderland, the Netherlands.

## Supplementary figures


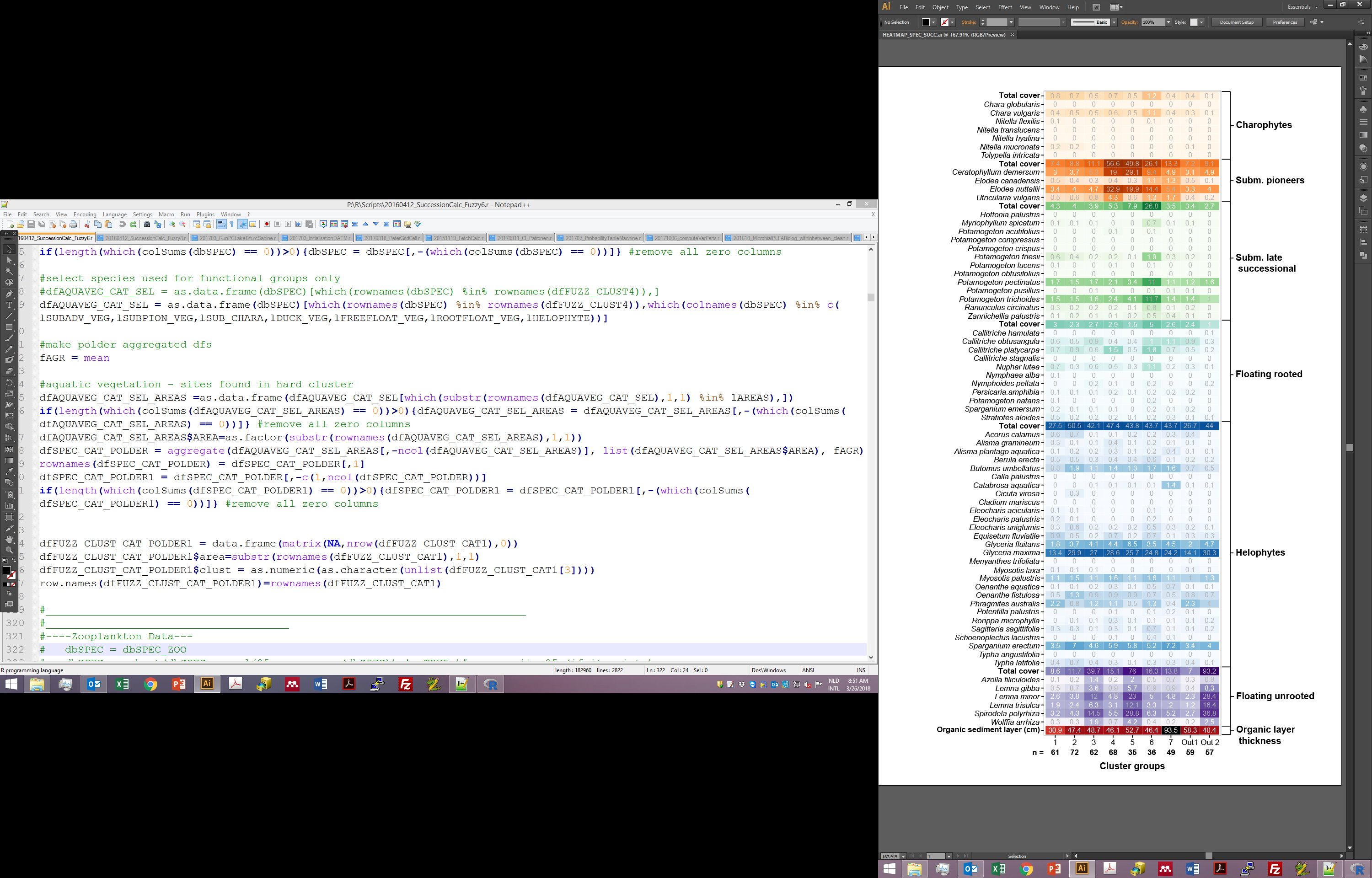


**Figure S1:** Heatmap of clusters (columns) and ranked along the successional gradient (except for groups 8 and 9; see text). Species (rows) are grouped by functional type (colours). Numbers in cells and colour intensity represent the relative cover (%) averaged over sites within clusters. Total cover: sum of cover of all species belonging to specified functional groups. Clusters Out1 and Out2 were not considered as part of the natural progression of succession and disregarded in further analyses.


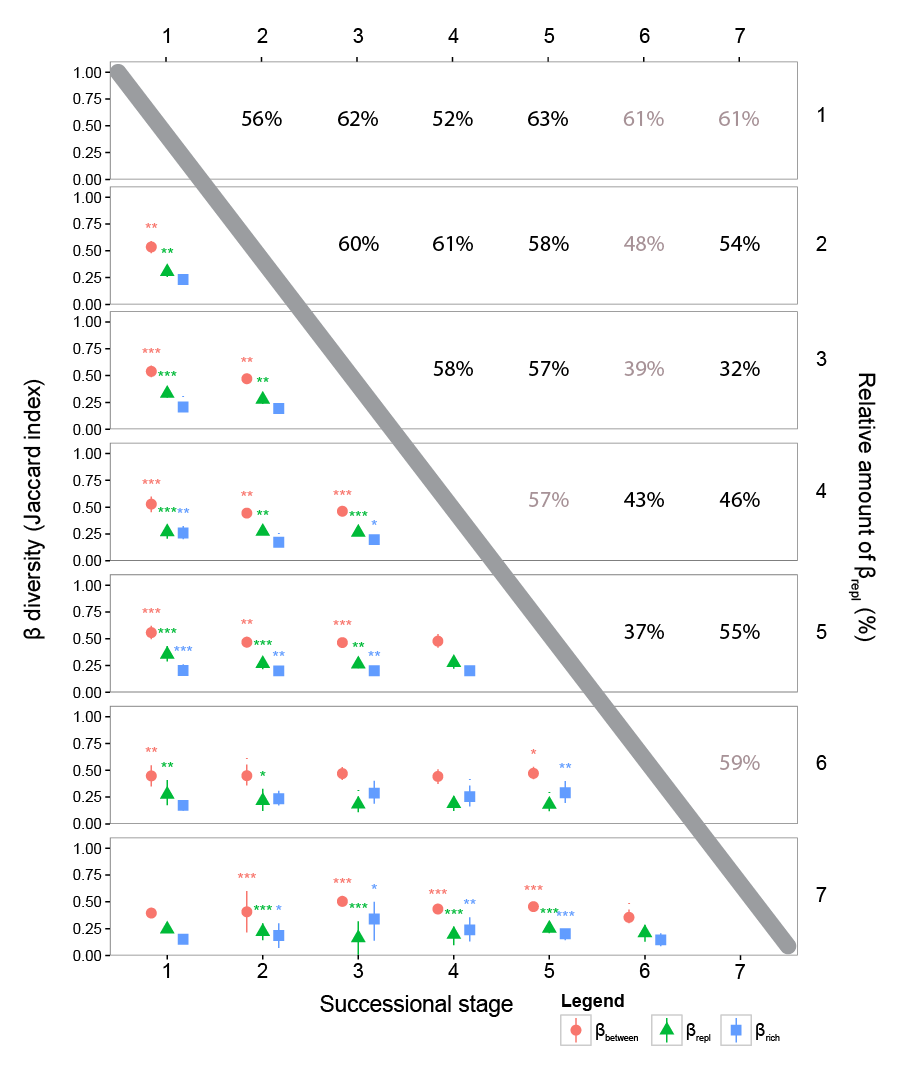


**Figure S.2:** β-diversity (expressed as a multi-site Jaccard dissimilarity index) between communities of different successional stages (β_between_) and its partitions of species replacement (β_repl_) and richness difference patterns (β_rich_) for presence/absence data. All possible pairwise combinations among successional stages (1-7) are shown. Significance against random difference in community composition based on a dbRDA analysis is indicated using symbols (***:p<0.001, **:p<0.01, *:p<0.05, ▪: p<0.10) and error bars give twice standard error around the polder mean. The values given in the upper triangle refer to the mean percentage of β-diversity which may be attributed to replacement. Values given are given in grey when the dbRDA model was not significant.


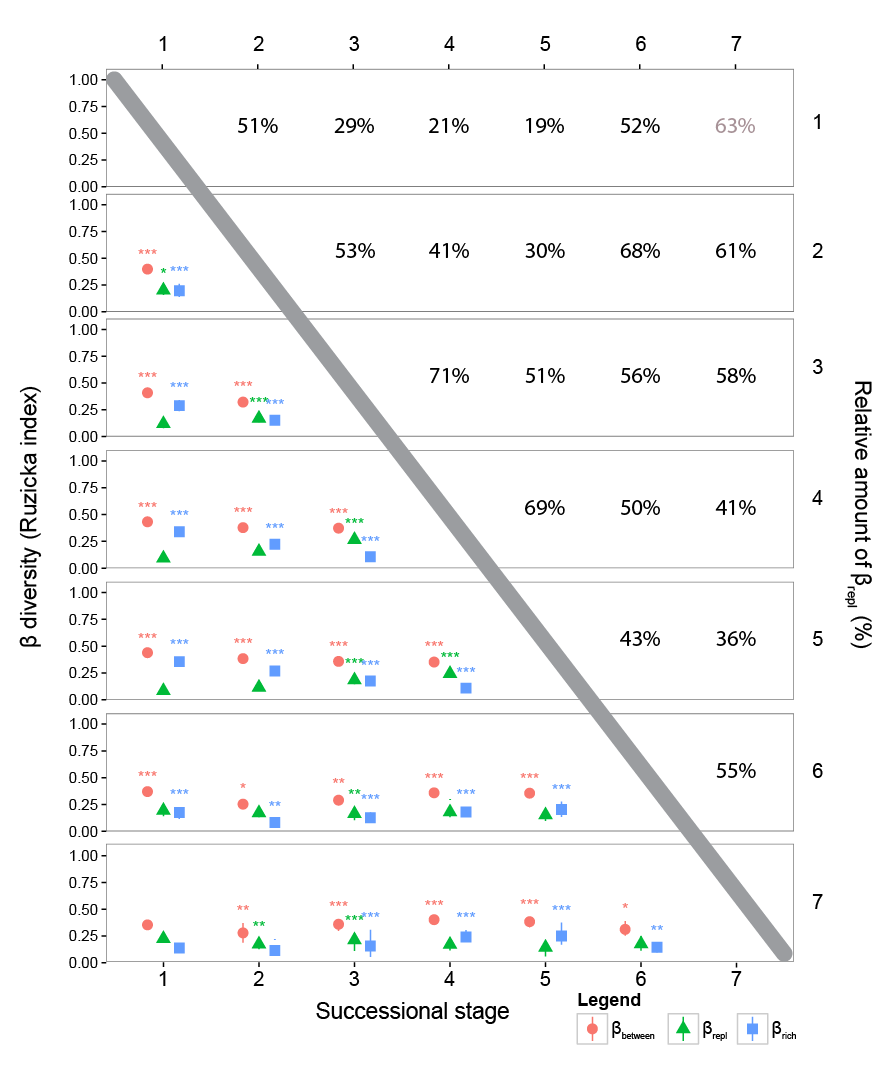


**Figure S3:** β-diversity (expressed as the total variance of a Ruzicka index distance matrix) between communities of different successional stages (β_between_) and its partitions of species replacement (β_repl_) and richness difference patterns (β_rich_) for presence/absence data. All possible pairwise combinations among successional stages (1-7) are shown. Significance against random difference in community composition based on a dbRDA analysis is indicated using symbols (***:p<0.001, **:p<0.01, *:p<0.05, ▪: p<0.10) and error bars give twice standard error around the polder mean. The values given in the upper triangle refer to the mean percentage of β-diversity which may be attributed to replacement. Values given are given in grey when the dbRDA model was not significant.


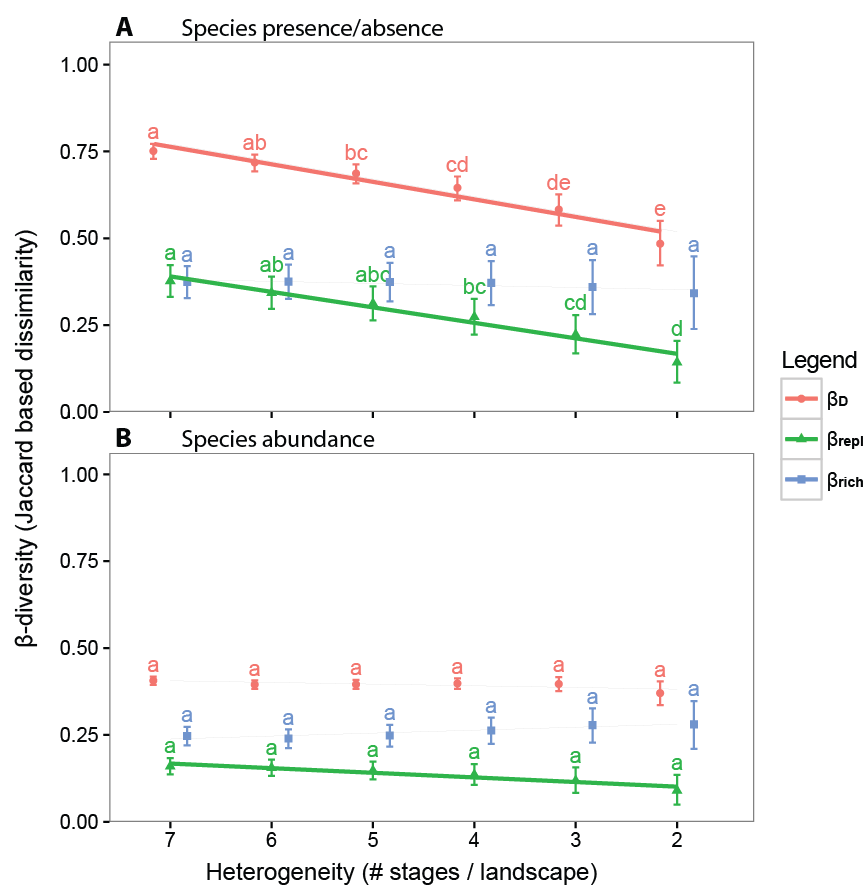


**Figure S4:** Effects of decreasing landscape-wide successional stage heterogeneity on the between successional β-diversity (β_D_) and its partitions (β_repl_, β_rich_) based on a scenario of No Management (scenario 2). (a) Changes in diversity based on species incidence data using a multi-site Jaccard dissimilarity index. (b) Changes in diversity based on species abundance data using the total variance of a Ruzicka (abundance based Jaccard) dissimilarity matrix. Error bars show the 97.5% confidence limits around the landscape-wide mean diversity values based on 21 simulated landscapes with 12 sites per landscape. Letters indicating pairwise significance between successional group means. Lines show significant trends (p<0.05) along the gradient of management based on a linear model and dashed lines show marginally significant trends (p<0.10).


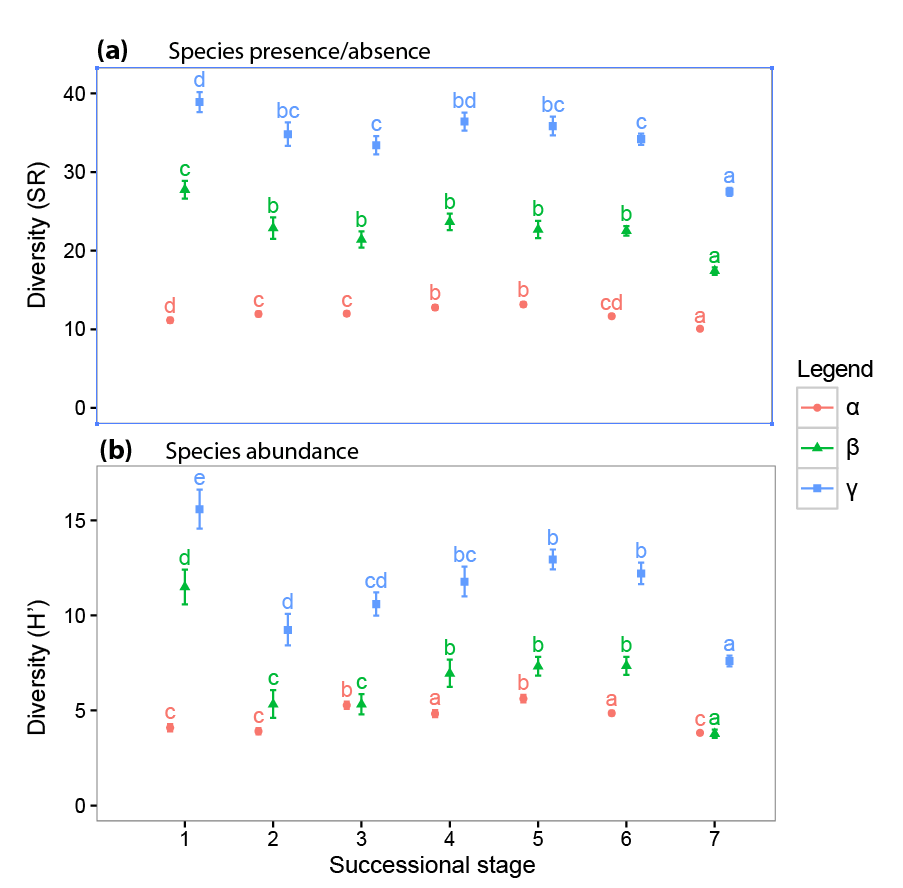


**Figure S5:** Diversity of successional stages in isolation, based on simulated landscapes of 12 randomly selected sites of the given successional stage. Twice the standard error around the landscape-wide mean diversity values based on 21 simulated landscapes. Letters indicating significance (p<0.05) of post-hoc comparisons between successional stages in terms of their diversity indeces.

# Supplementary tables

**Table S1:** Landscape coordinates (WGS84) in degrees.

| **Landscape number** | **Latitude (degrees)** | **Longitude (degrees)** |  |
| --- | --- | --- | --- |
| 1 | 52.12791 | 4.92838 |  |
| 2 | 52.04209 | 4.78927 |  |
| 3 | 51.88817 | 4.78154 |  |
| 4 | 51.98641 | 4.79103 |  |
| 5 | 51.85967 | 4.74393 |  |
| 6 | 52.16753 | 5.12449 |  |
| 7 | 51.88792 | 4.75494 |  |
| 8 | 51.86355 | 4.82294 |  |
| 9 | 51.96902 | 4.85145 |  |
| 10 | 52.04824 | 4.83968 |  |
| 11 | 52.05982 | 4.76403 |  |
| 12 | 51.91468 | 4.68527 |  |
| 13 | 52.11776 | 4.61890 |  |
| 14 | 51.91930 | 4.89699 |  |
| 15 | 51.93908 | 4.72608 |  |
| 16 | 51.75151 | 4.53776 |  |
| 17 | 52.25942 | 5.00921 |  |
| 18 | 52.19264 | 5.03050 |  |
| 19 | 52.27827 | 5.13003 |  |
| 20 | 51.93984 | 4.77225 |  |
| 21 | 52.03339 | 4.78264 |  |

**Table S2:** Conversion of Tansley abundance classes to numeric classes and percentage cover.

| Tansley score | Tansley numeric score | Cover  (%) |
| --- | --- | --- |
| *d* | 11 | 60 |
| *cd* | 10 | 40 |
| *ld* | 9 | 22 |
| *a* | 8 | 15 |
| *la* | 7 | 9 |
| *f* | 6 | 8 |
| *lf* | 5 | 3 |
| *o* | 4 | 2 |
| *lo* | 3 | 1 |
| *r* | 2 | 0.5 |
| *s* | 1 | 0.1 |

**Table S3:** Summary table of the different regression models and their parameters showing the trends present in the scenario analyses outlined in Figure 4.

| **Partition** | **Scenario** | **q** | **parameter** | **Value** | **±** | **se** | **DF** | **t-value** | **p-value** |
| --- | --- | --- | --- | --- | --- | --- | --- | --- | --- |
| *α* | *1: Selective management* | *SR* | intercept | 12.31 | ± | 0.18 | 125 | 67.52 | 7.82E-93 |
|  |  |  | slope | -0.13 | ± | 0.04 | 125 | -3.24 | 0.019 |
|  |  |  | adjusted R² | 8.35 |  |  |  |  |  |
|  |  | *H'* | intercept | 5.17 | ± | 0.12 | 125 | 44.83 | 1.72E-71 |
|  |  |  | slope | -0.15 | ± | 0.03 | 125 | -5.89 | 1.73E-05 |
|  |  |  | adjusted R² | 20.29 |  |  |  |  |  |
|  | *2: No management* | *SR* | intercept | 12.28 | ± | 0.40 | 125 | 35.91 | 2.07E-59 |
|  |  |  | slope | -0.28 | ± | 0.04 | 125 | -7.12 | 1.61E-07 |
|  |  |  | adjusted R² | 26.18 |  |  |  |  |  |
|  |  | *H'* | intercept | 5.10 | ± | 0.26 | 125 | 23.99 | 4.70E-41 |
|  |  |  | slope | -0.15 | ± | 0.03 | 125 | -5.82 | 9.90E-06 |
|  |  |  | adjusted R² | 19.32 |  |  |  |  |  |
| *β* | *1: Selective management* | *SR* | intercept | 22.91 | ± | 0.62 | 125 | 37.26 | 5.79E-63 |
|  |  |  | slope | 0.53 | ± | 0.14 | 125 | 3.88 | 0.005 |
|  |  |  | adjusted R² | 10.95 |  |  |  |  |  |
|  |  | *H'* | intercept | 6.68 | ± | 0.45 | 125 | 15.04 | 6.32E-25 |
|  |  |  | slope | 0.40 | ± | 0.10 | 125 | 4.02 | 0.003 |
|  |  |  | adjusted R² | 10.69 |  |  |  |  |  |
|  | *2: No management* | *SR* | intercept | 24.08 | ± | 1.17 | 125 | 26.02 | 6.22E-45 |
|  |  |  | slope | -0.89 | ± | 0.11 | 125 | -7.81 | 2.48E-08 |
|  |  |  | adjusted R² | 30.06 |  |  |  |  |  |
|  |  | *H'* | intercept | 7.91 | ± | 0.70 | 125 | 16.46 | 2.65E-25 |
|  |  |  | slope | -0.51 | ± | 0.07 | 125 | -7.46 | 4.45E-07 |
|  |  |  | adjusted R² | 27.91 |  |  |  |  |  |
| *γ* | *1: Selective management* | *SR* | intercept | 35.22 | ± | 0.67 | 125 | 53.13 | 2.78E-81 |
|  |  |  | slope | 0.40 | ± | 0.15 | 125 | 2.71 | 0.050 |
|  |  |  | adjusted R² | 6.76 |  |  |  |  |  |
|  |  | *H'* | intercept | 11.78 | ± | 0.47 | 125 | 24.98 | 3.38E-44 |
|  |  |  | slope | 0.04 | ± | 0.10 | 125 | 0.34 | 0.494 |
|  |  |  | adjusted R² | 1.79 |  |  |  |  |  |
|  | *2: No management* | *SR* | intercept | 36.36 | ± | 1.31 | 125 | 34.06 | 1.92E-57 |
|  |  |  | slope | -1.17 | ± | 0.13 | 125 | -9.12 | 6.72E-11 |
|  |  |  | adjusted R² | 0.37 |  |  |  |  |  |
|  |  | *H'* | intercept | 12.45 | ± | 0.86 | 125 | 19.34 | 4.25E-32 |
|  |  |  | slope | -0.58 | ± | 0.08 | 125 | -6.93 | 9.35E-07 |
|  |  |  | adjusted R² | 25.17 |  |  |  |  |  |

**Table S4:** Summary table of the different regression models and their parameters showing the trends present in the scenario analyses outlined in Figure 5.

| **Partition** | **q** | **parameter** | **Value** | **±** | **se** | **DF** | **t-value** | **p-value** |
| --- | --- | --- | --- | --- | --- | --- | --- | --- |
| *β_between D_* | *SR* | intercept | 0.77 | ± | 0.04 | 102 | 26.69 | 7.79E-41 |
|  |  | slope | -0.05 | ± | 0.00 | 102 | -11.52 | 2.2E-14 |
|  |  | adjusted R² | 51.90 |  |  |  |  |  |
|  | *H'* | intercept | 0.41 | ± | 0.02 | 102 | 21.52 | 1.53E-33 |
|  |  | slope | 0.00 | ± | 0.00 | 102 | -2.24 | 0.123656 |
|  |  | adjusted R² | 6.38 |  |  |  |  |  |
| *β_between repl_* | *SR* | intercept | 0.39 | ± | 0.06 | 102 | 12.71 | 3.80E-17 |
|  |  | slope | -0.04 | ± | 0.01 | 102 | -7.77 | 1.41E-07 |
|  |  | adjusted R² | 33.76 |  |  |  |  |  |
|  | *H'* | intercept | 0.17 | ± | 0.03 | 102 | 7.92 | 1.28E-07 |
|  |  | slope | -0.01 | ± | 0.00 | 102 | -3.91 | 1.17E-02 |
|  |  | adjusted R² | 13.25 |  |  |  |  |  |
| *β_between rich_* | *SR* | intercept | 0.38 | ± | 0.07 | 102 | 5.84 | 3.90E-05 |
|  |  | slope | -0.01 | ± | 0.01 | 102 | -0.79 | 4.01E-01 |
|  |  | adjusted R² | 0.03 |  |  |  |  |  |
|  | *H'* | intercept | 0.24 | ± | 0.04 | 102 | 3.99 | 4.76E-03 |
|  |  | slope | 0.01 | ± | 0.00 | 102 | 1.84 | 1.97E-01 |
|  |  | adjusted R² | 5.64 |  |  |  |  |  |
